# A Hybrid Deep Learning Approach: Integrating Short-Time Fourier Transform and Continuous Wavelet Transform for Improved Pipeline Leak Detection

**DOI:** 10.3390/s23198079

**Published:** 2023-09-25

**Authors:** Muhammad Farooq Siddique, Zahoor Ahmad, Niamat Ullah, Jongmyon Kim

**Affiliations:** 1 Department of Electrical, Electronics and Computer Engineering, University of Ulsan, Ulsan 44610, Republic of Korea; mfarooq229@mail.ulsan.ac.kr (M.F.S.); zahooruou@mail.ulsan.ac.kr (Z.A.); niamat016@mail.ulsan.ac.kr (N.U.); 2 PD Technology Co., Ltd., Ulsan 44610, Republic of Korea

**Keywords:** pipeline leak detection, short-time Fourier transform, continuous wavelet transform, principal component analysis, artificial neural network

## Abstract

A hybrid deep learning approach was designed that combines deep learning with enhanced short-time Fourier transform (STFT) spectrograms and continuous wavelet transform (CWT) scalograms for pipeline leak detection. Such detection plays a crucial role in ensuring the safety and integrity of fluid transportation systems. The proposed model leverages the power of STFT and CWT to enhance detection capabilities. The pipeline’s acoustic emission signals during normal and leak operating conditions undergo transformation using STFT and CWT, creating scalograms representing energy variations across time–frequency scales. To improve the signal quality and eliminate noise, Sobel and wavelet denoising filters are applied to the scalograms. These filtered scalograms are then fed into convolutional neural networks, extracting informative features that harness the distinct characteristics captured by both STFT and CWT. For enhanced computational efficiency and discriminatory power, principal component analysis is employed to reduce the feature space dimensionality. Subsequently, pipeline leaks are accurately detected and classified by categorizing the reduced dimensional features using t-distributed stochastic neighbor embedding and artificial neural networks. The hybrid approach achieves high accuracy and reliability in leak detection, demonstrating its effectiveness in capturing both spectral and temporal details. This research significantly contributes to pipeline monitoring and maintenance and offers a promising solution for real-time leak detection in diverse industrial applications.

## 1. Introduction

Pipelines play a crucial role in the distribution and transportation of liquid and gas materials over long distances. To ensure their efficient operation, it is essential to maintain high-quality standards and safety. However, current pipeline networks suffer from significant leakage issues, resulting in waste of natural resources. These leaks have severe consequences for the environment, human safety, property, and business reputations, causing financial losses owing to fines and cleanup expenses [1]. Saleh et al. [2] showed that many countries heavily rely on long-distance transportation of oil and water from desalination plants. However, approximately 60% of water resources are wasted each year owing to pipeline leaks [3]. To address this problem and achieve optimal pipeline performance, an effective leak detection system (LDS) is crucial. A reliable LDS should have the ability to promptly detect leaks, accurately locate them, minimize false alarms, easily integrate into existing systems, function well under various operating conditions, and utilize dependable sensors with low maintenance requirements [4,5]. An approach is proposed herein that combines acoustic emission (AE) technology and machine learning (ML) algorithms to develop an efficient and effective LDS.

Extensive research has been conducted on pipeline leak detection, with various aspects such as vision, sensors, transient response analysis, models, and data serving as the foundation for pipeline health management [6]. Recently, the predominant emphasis has been on feature extraction and use of AI-based recognition models in the domain of pipeline health diagnosis. These sophisticated models leverage the application of AI and ML methodologies to analyze AE data, facilitating the identification of discernible patterns that indicate the existence of a pipeline leak [7].

Many studies have used diverse methods to enhance the detection of pipeline leaks. Elforjani et al. [8] employed AE technology to identify the onset of fractures in pipelines. Banjara et al. [9] utilized waveform indicators extracted from AE data and subsequently employed support vector machines (SVMs) and relevance vector machines (RVMs) to detect and identify pipeline leaks. Rai et al. [10] developed a comprehensive pipeline health index by integrating multiscale analysis and the Kolmogorov–Smirnov (KS) test. Additionally, they integrated a Gaussian mixture model to assess the severity of pipeline leaks. Kim et al. [11] devised a pipeline leak indicator by analyzing the characteristics of acoustic emission waveforms and applying a two-sample KS test. Their results showed improved performance compared to the conventional feature-based indicators that are typically employed in leak detection. Meanwhile, Li et al. [12] developed a hybrid feature vector by combining AE time-domain characteristics and frequency-domain features. They utilized cross-entropy to extract discriminant features from the combined vector. These extracted features are employed with ANNs to significantly improve leak detection performance. Xu et al. [13] employed empirical mode decomposition (EMD) and continuous wavelet transform as time–frequency approaches to precisely identify leaks. Xu et al. [14] used variational mode decomposition (VMD) to denoise the AE signal and extracted Mel frequency cepstral coefficients (MFCCs) for classification purposes using SVMs. Although the above studies demonstrated improvements in pipeline leak diagnosis, they had limitations.

Pipeline leaks pose significant risks and can have far-reaching consequences. They not only compromise the structural integrity of the material, but they may also engender various forms of damage, such as fatigue rupture, stress cracks, corrosion cracks, and structural discontinuities. These issues can further disrupt the smooth flow of fluids or gases within the pipeline network, potentially leading to environmental hazards, safety concerns, and costly repairs. Effective leak detection and prevention measures are crucial for mitigating these issues and ensuring the safe and efficient operation of pipelines [15]. Despite these disruptions, the fluid’s intrinsic properties, such as intramolecular interactions and chemical bonding, may contribute to maintaining a relatively consistent flow under certain conditions [16].

To effectively address the potential risks associated with pipeline leaks, modern technology employs AE monitoring. This innovative approach relies on the detection of transient sound waves generated by the rapid release of energy due to changes in the material’s structural integrity, as observed during pipeline leaks and other structural defects [17]. AE sensors strategically positioned along the pipeline can accurately capture and assess stress waves propagating through the walls, and the resultant AE signal manifests as detectable transients referred to as ‘hits’ or ‘AE events’ [18]. However, to extract meaningful and actionable insights from the AE signal, crucial AE parameters, such as rise time, decay time, and event counts, must be isolated from the constant background noise inherent in the monitoring process [19]. Thus, the challenging aspect of AE technology is determining an optimal threshold level that effectively differentiates between pertinent AE signals and ambient noise. Accordingly, the occurrence of false alarms, which could otherwise lead to unnecessary maintenance or disruptive shutdowns, could be minimized. This intricate process of setting the threshold often necessitates the involvement of domain experts who possess the necessary experience and expertise to ensure accurate and reliable AE feature extraction. By effectively navigating these challenges, AE technology proves to be an invaluable tool for timely and precise detection of pipeline leaks, enabling proactive maintenance and risk mitigation measures to uphold the integrity, safety, and efficiency of pipeline systems.

The presence of noise in the AE signal can lead to false alarms if the threshold for feature extraction is set too low [20]. Additionally, feature extraction from AE signals requires expertise and subject-specific knowledge. The use of EMD for mode extraction can introduce extreme interpolation and mode mixing, adding complexity to the analysis [13]. Continued research aims to address these challenges and further enhance the accuracy and reliability of pipeline leak detection methods. The goal is to develop robust techniques that can effectively distinguish leak-related patterns from noise and improve the overall performance of leak detection systems. Deep learning (DL) techniques, specifically convolutional neural networks (CNNs) and convolutional autoencoders (CAEs), have shown promise in automating leak detection in pipeline infrastructures [5]. By extracting discriminant information from acoustic images using CNNs, the state of pipelines can be accurately classified. Additionally, CAEs can compress images and identify abnormalities, making them valuable for detecting leaks.

Recent efforts have enhanced leak detection accuracy using continuous wavelet transform (CWT). Ahmad et al. [21] introduced a reliable pipeline leak detection technique based on AE signals. The method involves the application of CWT to generate AE images that portray time–frequency scales, effectively capturing leak-related information through high-energy representations. These scalograms undergo processing through a CAE and an ANN to extract both global and local features. These features are then merged into a unified feature vector to enhance the accuracy and reliability of leak detection. A shallow ANN is employed to classify the pipeline leak state, achieving high accuracy across leak sizes and fluid pressures, as validated on an industrial pipeline testbed dataset. CWT scalograms were used by Sajjad et al. [21] to enhance the overall performance of leak detection. However, relying solely on CWT for pipeline leak detection using AE signals may have weaknesses. These methods exhibit a trade-off between time and frequency resolution, limiting their ability to simultaneously capture both temporal and spectral details [22]. The high dimensionality of CWT scalograms results in computational challenges, especially with large datasets. Additionally, the choice of wavelet function and parameters is crucial, as an inappropriate selection may result in suboptimal leak detection performance [23].

Addressing these weaknesses is essential for improving the accuracy, efficiency, and robustness of pipeline leak detection. To this end, the integration of complementary techniques and the development of hybrid approaches are often undertaken. For this purpose, the method proposed herein introduces a hybrid approach combining the features extracted from STFT and CWT for pipeline leak detection using AE signals. Unlike previous methods that rely solely on one transform technique, the integration of STFT and CWT enables a more comprehensive analysis of the AE signals, capturing both temporal and spectral information. STFT is employed to transform the time-domain AE signals into time–frequency representations. Accordingly, the energy distribution and spectral content are identified at different time intervals [24]. Meanwhile, CWT provides a localized and transient view of the signals, capturing fine details and variations over different scales [20]. By combining the features derived from STFT and CWT, the proposed method achieves a more holistic representation of the AE signals, enhancing the ability to detect and differentiate between normal operating conditions and leak-related patterns. This integration of features from multiple domains provides more robust and accurate detection of pipeline leaks. The significance of the hybrid approach—which leverages the strengths of both STFT and CWT—is its ability to capture a broader range of information from AE signals. This comprehensive representation improves the discrimination between leak-related patterns and background noise or other non-leak signals, leading to more precise and reliable identification of pipeline leaks. The performance of the proposed leak-detection technique was verified using a metallic steel pipe. The main contributions and novelty of this study are summarized as follows:

- This study introduces a novel approach combining CWT and STFT to analyze AE signals, resulting in a comprehensive representation that harnesses the strengths of the two methods.
- A unique feature extraction technique is proposed that combines the features derived from CWT and STFT scalograms to capture a broader range of information, including global spectral content and localized transient details and leading to enhanced leak detection performance.
- The proposed method significantly improves leak discrimination by effectively distinguishing between leak-related patterns and background noise or signals. It thus results in more accurate and reliable identification of pipeline leaks, even in challenging noise conditions.
- This study focuses on real-world applicability by testing the proposed leak-detecting technique on a metallic steel pipe. The feasibility and effectiveness of the proposed method in practical pipeline systems is demonstrated.

The remainder of this paper is structured as follows. Section 2 introduces the proposed technique, and Section 3 provides the technical background for the study. Section 4 details the data collection and experimental design for pipeline leakage detection. Section 5 examines the results of the proposed approach. Section 6 presents the conclusions of the study and notes future research directions.

## 2. Proposed Method

**Figure 1:**
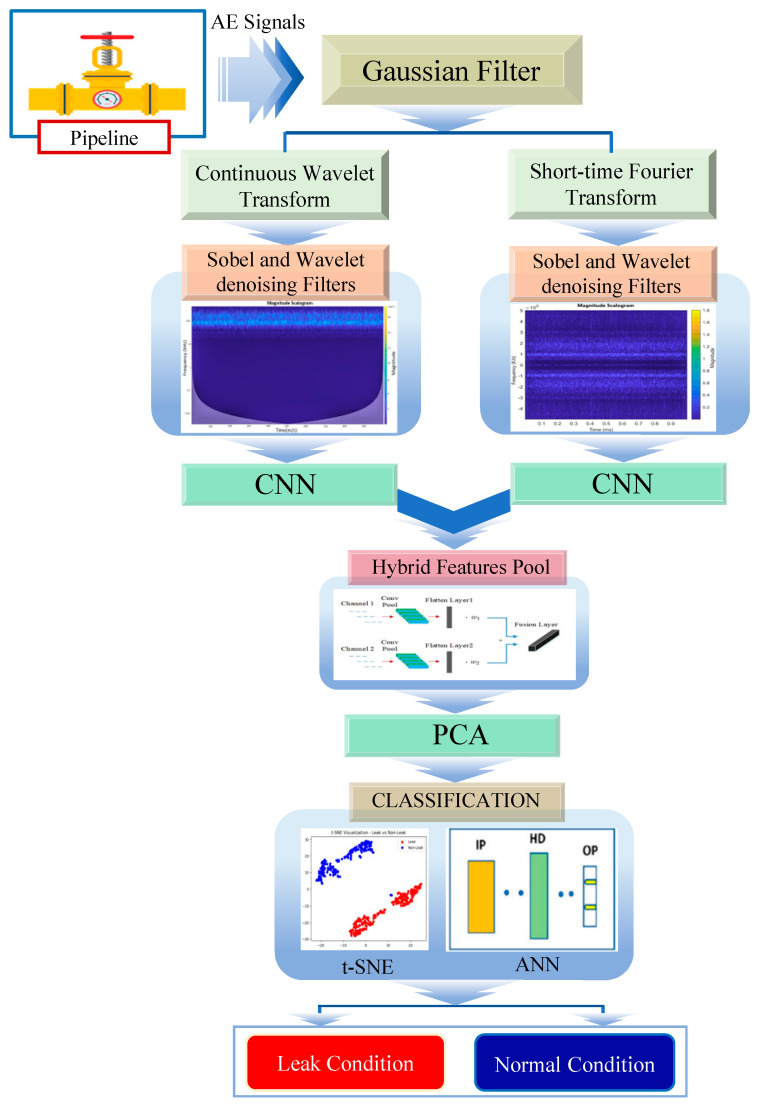
Comprehensive framework for pipeline leak detection.

The proposed method consists of sequential stages, as illustrated in Figure 1.

Step 1: AE signals are collected from the pipeline during both normal operating conditions and leak scenarios. Leaks in a pipeline result in the release of gas or fluid, creating stress waves that propagate through the pipeline’s surface toward the AE sensors. The AE sensors detect these stress waves for leak detection in the pipeline. To enhance the quality of the AE signals, a Gaussian filter is applied to remove undesirable noise.

Step 2: CWT and STFT are used to transform the Gaussian-filtered signals into scalograms. These scalograms visually depict energy level changes across time–frequency scales. They aid in leak detection by capturing localized transient details and global spectral content. The combination of CWT and STFT enhances the analysis of AE signals, making a valuable contribution to pipeline leak detection.

Step 3: After obtaining the scalograms from CWT and SFT, further processing is performed to enhance their quality. The scalograms are subjected to filtering using Sobel and wavelet denoising filters. These filters are employed to eliminate noise and undesirable artifacts from the images, leading to enhanced representations of the changes in energy levels over time and frequency. The filtering process significantly improves the visualization and analysis of the scalograms, facilitating more precise and dependable detection of pipeline leaks.

Step 4: The filtered scalograms from CWT and SFT serve as inputs to CNN for feature extraction. CNN applies its spatial pattern recognition and hierarchical learning capabilities to the scalograms. The resulting features from the STFT and CWT scalograms are combined in a hybrid feature pool. Specifically, the global spectral information from STFT and localized transient details from CWT are used. This hybrid feature pool provides a comprehensive representation of the AE signals, improving leak detection performance and enabling more accurate and reliable identification of leaks.

Step 5: After feature extraction, principal component analysis (PCA) is used to reduce dimensionality by transforming high-dimensional space while preserving informative features. By eliminating redundancy, PCA enhances computational efficiency and reduces the overfitting risk. This step further refines the feature representation and prepares the data for subsequent classification tasks. Accordingly, it contributes to the overall performance and reliability of the pipeline leak detection system.

Step 6: After the dimensionality reduction step using PCA, the next phase involves classification of the feature vectors. In this study, two techniques, t-distributed stochastic neighbor embedding (t-SNE) and ANN, are employed for this purpose. T-SNE is utilized to visualize and analyze the high-dimensional feature vectors by mapping them to a lower-dimensional space, while maintaining the distances between data points to the greatest extent possible. This helps in identifying meaningful clusters and patterns within the feature space. Subsequently, the feature vectors are fed into an ANN, a powerful machine learning model, for classification. The ANN learns from the labeled training data and leverages its hidden layers and activation functions to make accurate predictions on unobserved test data. This combination of t-SNE and ANN enables the pipeline leak detection system to effectively classify the feature vectors by distinguishing between normal and leak conditions with high accuracy and robustness.

## 3. Technical Background

### 3.1. Gaussian Filter

The Gaussian filter is a linear filter utilized for image smoothing by applying a Gaussian function. The shape of the Gaussian function resembles a bell curve and is characterized by its mean and standard deviation. By adjusting the standard deviation, the level of smoothing applied to an AE signal can be controlled [25].

In the current study, the Gaussian filter is applied to AE signals for noise reduction. The Gaussian filter functions by convolving the AE signal with a Gaussian function, which assigns greater weights to neighboring signal values while gradually diminishing the weights for more distant values. Through this filtering process, minor, random fluctuations within the AE signal are smoothed, while the fundamental attributes of the signal are preserved. By manipulating the standard deviation of the Gaussian function, the degree of smoothing applied to the AE signal can be adjusted. The Gaussian filter is widely employed in AE signal processing to mitigate noise and enhance signal clarity, facilitating more precise analysis and interpretation of data.

### 3.2. Continuous Wavelet Transform (CWT)

CWT is a robust signal processing technique that is employed for investigating the correlation between two time-series signals in the context of pipeline leak detection using AE signals. CWT utilizes specialized mathematical functions known as wavelets, which possess localized properties in both the time and frequency domains [25], as depicted in Equation (1). Through the application of CWT, a detailed representation of the signals in the time–frequency domain is obtained.

(1) $${CWT}_{x}\left(\tau ,s\right)=\frac{1}{\sqrt{\left|s\right|}}\int_{-\infty }^{+\infty }X(t)(\psi )(\frac{t-\tau }{s})dt$$

**Figure 2:**
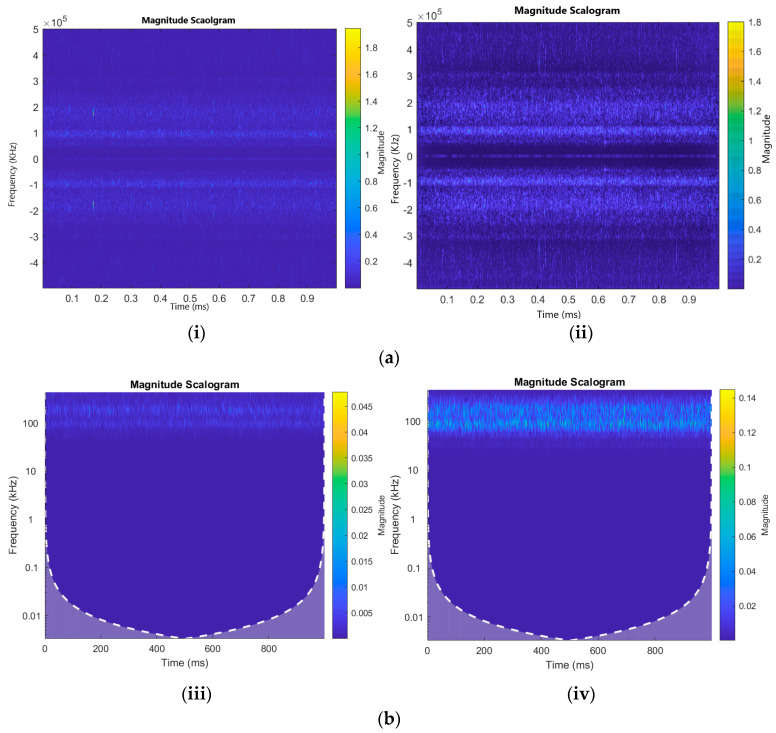
(**a**) STFT scalograms without (**i**) and with (**ii**) Sobel and Wavelet Denoising filters. (**b**) CWT scalograms without (**iii**) and with (**iv**) Sobel and Wavelet Denoising filters.

The CWT of a signal produces coefficients that are influenced by two parameters: scale (s) and translation (τ). Translation parameter τ represents the position of the wavelet in the time domain, while scale parameter s determines the central frequency and window length of the wavelet. When the scale is larger, it corresponds to lower frequencies, which enables the capture of global characteristics of the signal. Conversely, when examining smaller scales, the more complicated signal aspects become apparent as they align with higher frequencies. In this study, a CWT with source wavelet Morse (symmetry parameter = 3) is applied to the pipeline AE signals, and AE images are obtained. For complete details about Morse wavelet, readers are advised to refer to [26]. Figure 2a displays the scalograms, which are visual representations of the CWT coefficients.

### 3.3. Short-Time Fourier Transform (STFT)

STFT is a fundamental signal processing technique that is used in pipeline leak detection with AE signals. It reduces the AE signals to shorter segments and applies the FT to each segment. This offers a comprehensive representation of signals in the time–frequency domain, enabling analysis of their spectral content and temporal variations. STFT plays a crucial role in capturing both the frequency components and their temporal changes in the AE signals. This facilitates the identification of patterns associated with both leaks and normal operating conditions. By generating a spectrogram that visually represents the energy distribution across frequencies and time intervals, it becomes feasible to discern specific patterns and spectral characteristics linked to pipeline leaks. This utilization of STFT enhances the overall effectiveness and reliability of the leak detection system by extracting valuable information from the signals. Notable changes in the spectrogram, such as distinct frequency components or concentrated energy at specific time intervals, can serve as indicators of potential leak-related events, contributing to accurate detection of pipeline leaks [24]. Moreover, STFT facilitates investigation of the relationship between AE signals and pipeline leaks. Researchers can analyze the spectrogram to isolate time intervals where the spectral properties of the signal diverge from the background, indicating possible leak occurrences. The identification of significant frequency components or concentrated energy regions in the spectrogram provides valuable insights into the occurrence and attributes of pipeline leaks. Through the examination of these features, researchers can obtain crucial information about the timing and characteristics of the leaks. 

Additional techniques, such as applying window functions and overlapping during STFT computation, can be employed to improve resolution and reduce spectral leakage artifacts. Incorporating STFT in pipeline leak detection facilitates comprehensive exploration of the spectral characteristics and temporal variations, enhancing the accuracy, reliability, and effectiveness of leak detection techniques that rely on AE signals. By leveraging the advantages of STFT in this domain, it becomes possible to make informed decisions and take proactive measures to ensure the integrity and safety of pipelines in various industrial applications.

### 3.4. Sobel and Wavelet Denoising Filters

The Sobel edge filter is a widely used digital filter in image processing that specializes in detecting edges within images. By convolving an image with a small matrix known as the Sobel operator, this filter calculates the gradient at each pixel to enhance the edges present in the image [27]. When applied to CWT and STFT scalograms, which represent the time–frequency information of a two-dimensional signal, the Sobel filter amplifies edge details in the scalograms. By examining the neighboring frequency components within the scalogram, the Sobel filter can identify abrupt changes in the signal’s frequency content that often correspond to significant events or features in the signal, such as transients or anomalies. This makes the Sobel edge filter a valuable tool for edge detection in numerous image processing applications [28]. 

Wavelet denoising filters are image processing techniques that are utilized to enhance the quality of scalograms by reducing noise. These filters leverage the properties of wavelets, which are mathematical functions localized in both time and frequency domains. The denoising process involves decomposing the scalograms into wavelet coefficients to represent their details at different scales and positions. Thresholding techniques are then applied to these coefficients to selectively remove or attenuate noise while preserving important signal features. This objective can be achieved through soft or hard thresholding methods. Soft thresholding attenuates small coefficients, whereas hard thresholding sets small coefficients to zero. In the current study, a soft thresholding denoising filter was used because it is more effective in reducing noise while preserving important signal features [29]. By reconstructing the scalogram using the modified coefficients, wavelet denoising filters effectively reduce noise and improve scalogram quality [30].

In this study, both the Sobel and wavelet denoising filters were applied to images generated through CWT and STFT. These filters enhance the quality and clarity of the images by emphasizing edges and reducing noise. The Sobel filter enhances the edges of both scalograms, allowing better detection and visualization of correlated regions [31]. On the other hand, the wavelet denoising filter helps remove noise from both CWT and STFT scalograms, resulting in a cleaner representation of the signal’s time–frequency characteristics. This study aimed to enhance the precision and comprehensibility of the analysis findings by implementing these filters. The following figures illustrate the comparison of these filters. Figure 2a depicts the STFT comparison while using Sobel and wavelet denoising filters. Figure 2b shows the comparison between Sobel and wavelet denoising with and without filtered scalograms.

### 3.5. Convolutional Neural Networks (CNNs)

CNNs are a type of deep learning model designed for analyzing visual data such as images. CNNs leverage the concept of local receptive fields and shared weight parameters to efficiently extract hierarchical features from input data. Comprising multiple convolutional layers followed by pooling and fully connected layers, CNNs can automatically learn and represent complex patterns and structures in images. By applying convolutional filters to localized regions of the input, CNNs can capture spatial relationships and hierarchically build representations from low-level to high-level features [17].

In the pipeline leak detection study, CNNs were applied to each of the scalograms derived from the CWT and STFT of the acoustic emission signals. The scalograms provided a visual representation of the energy distribution across different time–frequency scales and highlighted leak-related patterns. The scalograms derived from STFT or CWT were utilized as inputs to CNNs. The CNNs demonstrated their ability to automatically extract pertinent features and analyze the spatial and frequency characteristics of the scalograms in a hierarchical manner. This capability allowed the CNNs to capture both local and global patterns in the acoustic emission data, enabling accurate detection of pipeline leaks. CNNs are an integral part of the pipeline leak detection algorithm as they are utilized to extract features effectively from the visual representations of acoustic emission signals known as scalograms [32]. By training the CNNs on a large dataset, they learn to distinguish leak-related patterns from background noise, improving the accuracy and reliability of the system. Leveraging the capabilities of CNNs enables the development of a robust and automated approach for identifying and classifying leaks based on the information extracted from the scalograms. This utilization of CNNs contributes significantly to the overall success of the pipeline leak detection method.

Table 1 presents a comprehensive summary of the neural network model layers by listing their input shape, output shape, and the corresponding number of parameters. The model commences with Conv1, a convolutional layer that accepts an input of shape (3, 224, 224), generates an output of shape (16, 224, 224), and consists of 448 parameters with a rectified linear unit (ReLU) activation function. This is succeeded by a max-pooling layer that reduces the spatial dimensions by half. Subsequently, Conv2 operates on an input of shape (16, 112, 112), yielding an output of shape (32, 112, 112) with 4640 parameters, also utilizing ReLU activation. Another max-pooling layer further decreases the dimensions. Conv3 processes an input of shape (32, 56, 56), generating an output of shape (64, 56, 56) and utilizing 18,496 parameters with ReLU activation. Once again, the dimensions are reduced through a subsequent max-pooling layer. The output is reshaped into a flattened form (50,176) to be fed into the fully connected layers, FC1 and FC2. FC1 takes the flattened input and produces an output of shape (128) comprising 6,407,040 parameters with ReLU activation. Finally, FC2 generates the final output shape (10), utilizing 1290 parameters. This tabular representation provides a comprehensive overview of the layer configurations and parameter quantities, offering insights into the model structure and complexity with incorporation of the ReLU activation function.

### 3.6. Principal Component Analysis (PCA)

PCA is a robust statistical methodology employed in the analysis of data and machine learning to convert datasets with a high number of dimensions into a space with fewer dimensions. The primary objective of PCA is to capture the most significant patterns and variability within the data by identifying the principal components. These components are orthogonal linear combinations of the original features and are ranked based on the amount of variance they explain. By reducing the dimensionality of the dataset while preserving the most informative characteristics, PCA aids in simplifying complex data representations, enhancing interpretability, and improving computational efficiency [33].

In the present study focusing on pipeline leak detection, the application of PCA played a pivotal role in the feature extraction process. The initial step involved the generation of hybrid feature pools derived from the scalograms of both STFT and CWT. These feature pools captured essential temporal and spectral information from AE signals, which contained valuable energy changes related to pipeline leaks. To further optimize the feature representation, PCA was employed to select a reduced set of principal components that retained the most crucial leak-related information. By discarding less significant components, the dimensionality of the feature vectors was reduced, mitigating the risk of overfitting and improving the classification performance of the subsequent leak detection algorithm. The utilization of PCA in the pipeline leak detection study significantly enhanced the system’s overall effectiveness and accuracy. By integrating PCA-enhanced features with a combination of t-SNE and ANN classifiers, the leak detection algorithm demonstrated remarkable performance in distinguishing between normal and leak conditions. The selected principal components allowed the algorithm to focus on the most discriminative aspects of the AE signals, effectively separating leak-related patterns from background noise or non-leak events.

### 3.7. t-Distributed Stochastic Neighbor Embedding

t-distributed stochastic neighbor embedding (t-SNE) is a powerful technique used for exploratory data analysis and visualization. It excels at capturing intricate and nonlinear relationships and patterns in the data. The utilization of t-SNE facilitates the representation of complicated structures in a more comprehensible and meaningful manner by reducing the dimensionality of the data. This makes it a valuable tool for gaining insights, identifying clusters or patterns, and understanding the underlying relationships in the data. Whether dealing with intricate datasets or nonlinear structures, t-SNE is an effective method for exploring and visualizing complex data [34].

In the present research, t-SNE played a crucial role in improving the classification process of the pipeline leak detection system. After extracting hybrid features from the filtered scalograms of STFT and CWT, the resulting feature vectors contained important information about leak patterns and normal operating conditions. To enhance the accuracy of classification and gain a better understanding of the structure of the feature space, t-SNE was utilized to map the high-dimensional feature vectors to a lower-dimensional representation. This mapping enabled visualization and examination of the distribution and separability of feature clusters, facilitating identification of distinct patterns associated with leak and non-leak instances. By reducing the dimensionality while preserving the relationships between instances, t-SNE enhanced the classification algorithm’s ability to discriminate between leak-related patterns and background or non-leak signals. The resulting lower-dimensional representation provided a clearer visualization of the feature clusters, aiding in identification of anomalies and supporting decision-making processes. 

### 3.8. Artificial Neural Network (ANN)

ANN is a machine learning model composed of three fundamental layers: an input layer, a hidden layer, and an output layer [35]. In this study, the input layer of the ANN is responsible for receiving input features. Once collected, these features undergo transformations in the hidden layer through a series of matrix operations. The hidden layer’s role is to extract meaningful patterns and representations from the input data. Subsequently, the output layer employs an activation function, specifically the ReLU function, to categorize the transformed features based on their respective roles. The choice of ReLU as the activation function in the output layer introduces nonlinearity, allowing the neural network to learn complex relationships between the input features and their corresponding classes [36]. This enables the ANN to effectively model intricate connections and enhance its capacity to perform tasks such as classification, where nonlinear decision boundaries are common. 

ANN played a crucial role in the pipeline leak detection study as the final stage of classification, using the hybrid features extracted from STFT and CWT filtered scalograms. These feature vectors contained vital information related to AE patterns associated with leaks and normal operating conditions. By inputting these feature vectors into the ANN, it effectively distinguished between leak and non-leak instances, enabling accurate leak detection. During the training phase, the ANN learned from labeled examples, adjusting the connection weights to minimize classification errors and optimize predictive accuracy. Leveraging the nonlinear mapping capabilities of the ANN, it captured subtle leak-related patterns in the hybrid features. This robust and efficient classification system will contribute significantly to detecting leaks in real-world scenarios, enhancing safety and operational efficiency. The final step of the comprehensive framework for pipeline leak detection in Figure 1 for classification into leak and non-leak conditions is carried out by ANN.

## 4. Experimental Setup

**Figure 3:**
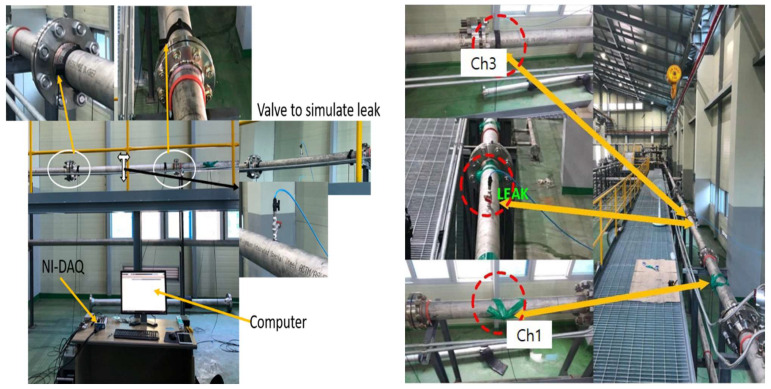
Acoustic emission leak detection: experimental setup and methodology.

**Figure 4:**
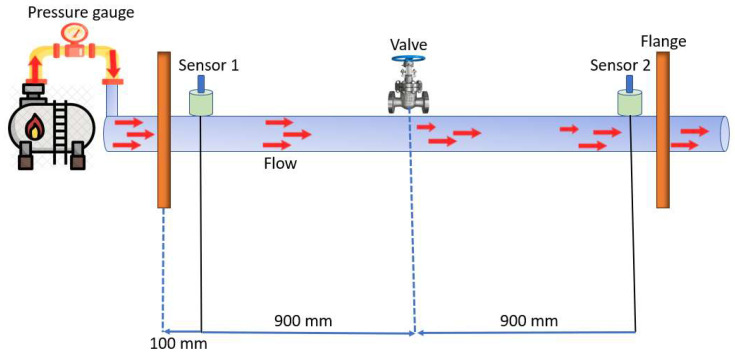
Schematic illustration of acoustic emission leak detection method.

The experimental setup employed in this study is illustrated in Figure 3 and Figure 4, along with the corresponding schematics. To simulate pipeline leaks, a stainless-steel water pipeline measuring 114.4 mm in outer diameter and 6 mm in thickness was fitted with AE R15I-AST sensors provided by Mistras Group, Inc (New Jersey, NJ, USA), to capture AE signals. The signals were recorded at a sampling frequency of 1 MHz using a National Instruments (Austin, TX, USA) data-collection apparatus (model NI-9223) connected to a computer. To replicate leaks of varying sizes, a hole was created in the pipeline using an electric drill. A fluid control valve was welded at the precise location of the hole to regulate fluid flow. For the experiment, water was chosen as the fluid owing to its non-toxic and non-hazardous properties. This selection allowed us to simulate controlled and secure leak scenarios. 

**Figure 5:**
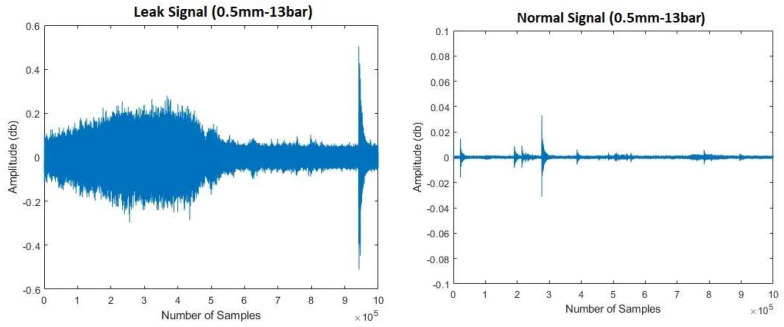
Investigating water leak detection: acoustic emission signals under leak and normal conditions (pressure = 13 bar).

**Figure 6:**
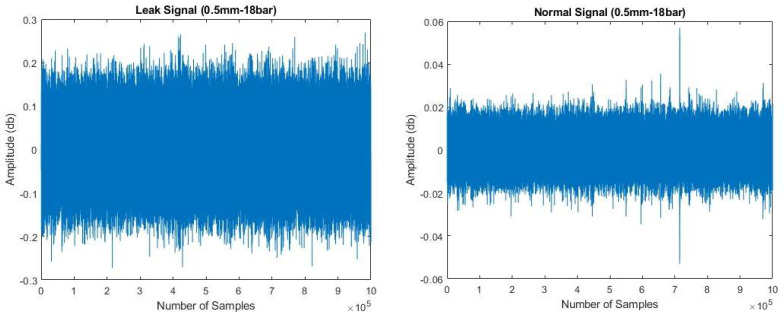
Investigating gas leak detection: acoustic emission signals under leak and normal conditions (pressure = 18 bar).

The leak simulation was executed through incorporation of a valve into the pipeline, followed by tests conducted under varying pressure conditions of 13 and 18 bar. The procedural steps commenced with the initial closure of the valve, during which data collection occurred for 2 min while the pipeline operated normally. Subsequently, the valve was opened to introduce a leak of 1 mm, and data collection was extended for an additional 4 min. Closure of the valve marked stabilization of the pipeline flow. This entire sequence was replicated for both pressure levels, maintaining the same size of the leak pinhole. In total, 360 signal samples were gathered for each test, with 120 samples stemming from the normal operational state and 240 samples arising from the leak condition. These samples were then utilized for subsequent analysis. Figure 5 and Figure 6 depict the AE signals obtained during the leak and normal operation of the pipeline, respectively. To ensure the safety of personnel, the fluid leaking from the pipeline was collected in a container using a hose.

### 4.1. Proposed Method: Surveillance Zone Identification

The evaluation of the surveillance zone, as outlined in ISO standard 18211:2016, involves the assessment of the attenuation characteristics of acoustic emission (AE) signals caused by the noise generated by the AE source. This evaluation is conducted before data collection from the AE sensor takes place. In the field of audio engineering, attenuation is a term used to describe the decrease in the intensity of a signal, typically quantified in decibels (dB). The attenuation characteristic of an acoustic emission (AE) sensor can be determined by utilizing the following Equation (2) [18].

(2) $$A_{dB}=20\mathrm{Log}\frac{V}{V*}$$

Equation (2) denotes the measured potential (V) and the reference potential (V∗). The term “measured AE potential” pertains to the acoustic emission (AE) signal acquired from the AE sensor. In the context of AE, the reference point of 0 dB corresponds to the AE signal potential of 1 µV at the AE sensor without any amplification.

**Figure 7:**
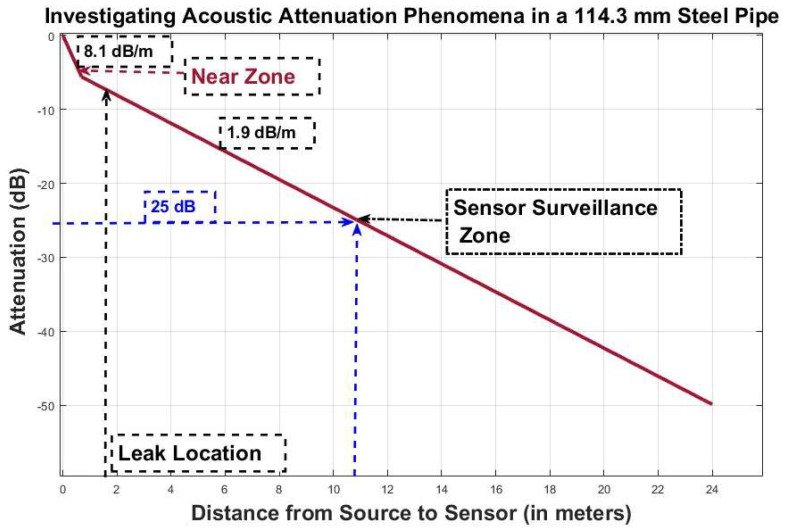
AE signal attenuation in 114.3 mm diameter steel pipe.

The present study employs the HSU-Nielsen test as an acoustic emission (AE) source in order to assess the attenuation characteristics of the AE sensor. The HSU-Nielsen test entails performing a pencil-led break test, wherein a lead with a diameter of 0.5 mm is applied to the pipeline’s surface to induce an acoustic emission event. The acoustic emission (AE) hits observed in the HSU-Nielsen test exhibit similarities to AE sources commonly associated with natural occurrences, such as leaks. Figure 7 illustrates the attenuation characteristics of an industrial pipeline that is filled with fluid and has an outer diameter of 114.3 mm. In order to ensure optimal performance of the AE sensor R15I-AST installed on the pipeline, it is recommended that the distance between two sensors falls within a specified range where the attenuation is maintained at a level below 25 dB. Based on the data presented in Figure 7, it can be observed that the specified level of attenuation is observed at a distance of 10.9 m. As a result, by installing an AE sensor R15I-AST on an industrial pipeline with an outer diameter of 114.3 mm, one can achieve effective surveillance coverage over a distance of 10.9 m. Once the attenuation of the acoustic signal exceeds 25 dB, the acoustic emissions (AE) become noticeable, registering at levels below 10% of the overall peak value of the AE signal. Consequently, distinguishing between the noise generated by leaks and the ambient noise becomes a challenging task. In this study, a method is proposed, which involves employing an adaptive threshold set at 10% of the peak value to differentiate between acoustic emission (AE) events and the surrounding background noise. By utilizing this approach, leaks can be effectively identified within a distance of 10.9 m or 10,900 millimeters, using only a single AE sensor, specifically the R15I-AST model.

### 4.2. Dataset Collection and Description

The experimental setup included a pipeline employed for conveyance of liquids and gases, wherein the pressure was controlled at either 13 or 18 bar using a centrifugal pump. Initially, the valve was kept closed, and the pipeline operated to move fluid under a pressure of 13 bar, capturing data under standard operational conditions. Subsequently, under the same pressure setting, the leak valve was gradually opened to 1 mm, leading to collection of data that constituted Dataset-1. After acquiring data at 13 bar pressure, the leak valve was closed, and the pipeline was set to transport fluid under a pressure of 18 bar to acquire data under normal conditions. Next, under the same pressure level, the leak valve was opened to 0.3 mm, resulting in the formation of Dataset-3. The same procedure was repeated to obtain Dataset-2 and Dataset-4. However, to ensure safety, the leak valve was restricted to a maximum opening of 0.5 mm during data collection for Dataset-2 and Dataset-4. A comprehensive listing of each data set is provided in Table 2.

## 5. Results and Discussion

The arrangement of training and testing data played a crucial role in assessing the effectiveness of the proposed method. In this study, the training phase involved the utilization of datasets comprising a 1 mm leak size at two fluid pressures: 13 bar and 18 bar. For evaluation purposes, three additional datasets were employed, each of a different leak size (1 mm, 0.5 mm, and 0.3 mm) and fluid pressure (13 bar and 18 bar). The overall dataset for this study comprised 960 normal samples and 960 leak samples, resulting in a total of 1920 samples. During the model training process, a common practice is to split the available dataset into separate training and testing sets. In the current study, a random division of the dataset was performed, with the allocation of 80% of the samples for training purposes and the remaining 20% for evaluating model performance. A rigorous testing procedure was implemented to ensure the credibility and consistency of the findings. The testing phase was conducted 10 times, corresponding to the number of experiments conducted. The purpose of repeating the testing phase multiple times was to gain a more thorough understanding of the model performance and to validate the obtained results. This approach was intended to minimize the potential influence of random variations and provide robust insights into model effectiveness and reliability.

### Evaluating Model Performance: A Comparative Analysis

The proposed approach employs CNN to extract features from two kinds of scalograms, i.e., CWT and STFT. These extracted features are merged into a feature vector that is fed into t-SNE and ANN to determine the pipeline conditions. The method was tested with both liquids and gases under various hole and pressure conditions, as outlined in Table 1. The performance was evaluated using key metrics of accuracy, precision, recall, and F1 score.

Accuracy indicates overall correctness by measuring the proportion of correctly classified samples among the total. Precision gauges the ratio of true positive predictions to total positive predictions, assessing the algorithm’s ability to identify positives correctly. Recall (sensitivity) measures the ratio of true positive predictions to actual positive samples, demonstrating the algorithm’s capture of positive instances. The F1 score combines precision and recall, offering a balanced performance assessment by considering false positives and negatives. These metrics collectively evaluate the classification algorithm’s efficacy and result reliability. Equations (3)–(6) provide specific formulas to quantitatively gauge the proposed method’s performance metrics in classifying different pipeline conditions. By comparing these metrics with a reference or baseline, we can determine the effectiveness and superiority of the proposed method.

(3) $$\mathrm{Accuracy}\;=\frac{\sum_{a}^{A}\;TP_{\alpha }}{N}$$

(4) $$Precision=\frac{\sum_{\alpha }^{A}\;n_{\alpha }\times \left(\frac{TP_{\alpha }}{TP_{\alpha }\;+\;FP_{\alpha }}\right)}{N},$$

(5) $$Recall=\frac{\sum_{\alpha }^{A}\;n_{\alpha }\times \left(\frac{TP_{\alpha }}{TP_{\alpha }\;+\;FN_{\alpha }}\right)}{N},$$

(6) $$F-1=\frac{1}{N}\sum_{\alpha }^{A}\;n_{\alpha }\times 2\times \sum_{\alpha }^{A}\;\left(\frac{{\;\mathrm{Recall}\;}_{\alpha }\times {\mathrm{Precisio}\;}_{\alpha }}{{\;\mathrm{Recall}\;}_{\alpha }+{\mathrm{Precisio}\;}_{\alpha }}\right)$$

The terms “TPa”, “FPa”, and “FNa” are, respectively, short for true-positive, false-positive, and false-negative outcomes related to class A. False-positive results are observed when samples are erroneously categorized as members of class A, even though they do not truly belong to class A. Conversely, true-positive outcomes signify the precise classification of samples that genuinely pertain to class A. False-negative findings pertain to instances erroneously classified into a different category, rather than category A, despite their actual membership in the A category. The variable $n_{\alpha }$ represents the collection of samples belonging to class A. The sum of TPa and FNa represents the aggregate count of samples that accurately pertain to class A, denoted as $n_{\alpha }$. In a similar vein, the sum of FPa and the discrepancy between the overall data sample count and $n_{\alpha }$ signifies the aggregate count of misclassified samples falsely attributed to class A. The variable N denotes the aggregate number of data samples contained in the testing sets utilized in the classification algorithm. 

To assess the effectiveness of the proposed model, a comparative analysis was undertaken with three other relevant models that have been previously utilized for similar purposes. The first two models are sub-parts of the proposed model and are named STFT-CNN and CWT-CNN models. The third one was proposed by Prosvirin et al. [37] for centrifugal pump fault detection and utilizes a combination of a CAE and neural network (NN). The focus of this model is detecting faults in centrifugal pumps, and it employs the kurtogram as a key feature extraction technique.

In the current study, a hybrid approach was designed that combines the features extracted from STFT and CWT for pipeline leak detection using AE signals. To compare the model, two sub-models, namely, STFT-CNN and CWT-CNN, were used to identify the performance parameters using the same dataset. First, using the STFT-CNN model, the accuracy, precision, F-1 score, and recall calculated were 93.64%, 93.67%, 93.64%, and 93.65%, respectively, as shown in Table 3. In the same manner, while using the CWT-CNN model under the same conditions, the respective average performance parameters were 95.57%, 96.50%, 95.11%, and 95.78%. The proposed hybrid model outperformed the sub-models in all performance parameters. The accuracy, precision, F-1 score, and recall were 99.22%, 99.84%, 99.34%, and 98.67%, respectively, as shown in Table 3, indicating a comprehensive representation that harnesses the strengths of both methods. The proposed method also offers a substantial enhancement in leak discrimination by effectively differentiating between patterns associated with leaks and background noise or signals. This leads to more precise and dependable detection of pipeline leaks, even in difficult noise conditions.

The final approach recommended by Prosvirin et al. [37] for comparative analysis involves kurtograms derived from preprocessing vibration signals. This method employs a deep architecture based on a CNN contractive autoencoder (CNN-CAE) to detect mechanical defects in the conditioned plant (CP). When we applied Prosvirin et al. [37] methodology to our dataset, we achieved performance metrics for accuracy, precision, recall, and F1 scores of 96.88%, 96.82%, 95.86%, and 95.86%, respectively. In contrast, our proposed model demonstrated respective metrics of 99.22%, 99.84%, 99.34%, and 98.67%, as shown in Table 3. However, the signals captured from the pipeline AE encompassed continuous background noise alongside AE hits related to leaks. While kurtograms and scalograms can capture variations in the signals, they are susceptible to the influence of strong background noise. 

The proposed hybrid model harnesses the strengths of both STFT-CNN and CWT-CNN models. It provides a more comprehensive representation of the AE signals, enabling improved discrimination between leak-related patterns and background noise. This preprocessing step plays a crucial role in enhancing the accuracy of our proposed method compared to the reference method, as it effectively mitigates the impact of strong background noise on the AE images. The combination of the hybrid STFT-CNN and CWT-CNN model features, along with the deep architecture based on a CNN-CAE, offers a novel and effective solution for pipeline leak detection. The proposed model not only surpasses the accuracy of the reference method, but also provides a more robust and reliable approach for identifying pipeline leaks in the presence of background noise. The table also shows a full summary of the performance parameters of the models.

**Figure 8:**
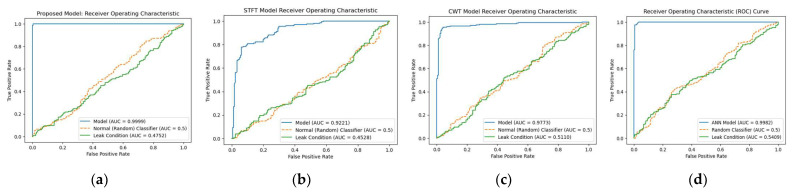
ROC curve comparison: (**a**) proposed model vs. (**b**) STFT-CNN, (**c**) CWT-CNN, and (**d**) Prosvirin et al. models (leak size = 1.0 mm).

**Figure 9:**
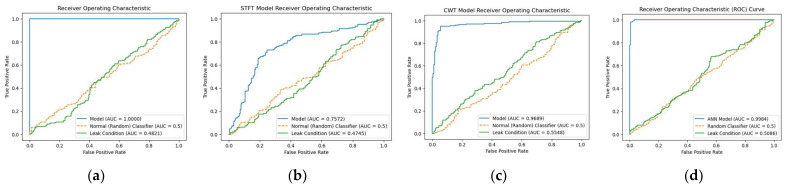
ROC curve comparison: (**a**) proposed model vs. (**b**) STFT-CNN, (**c**) CWT-CNN, and (**d**) Prosvirin et al. models (leak size = 0.5 mm).

**Figure 10:**
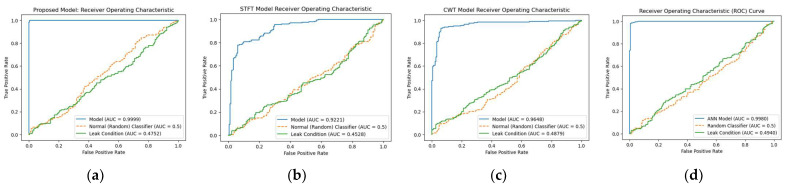
ROC curve comparison: (**a**) proposed model vs. (**b**) STFT-CNN, (**c**) CWT-CNN, and (**d**) Prosvirin et al. models (leak size = 0.3 mm).

The receiver operating characteristic (ROC) curve for the proposed model was calculated and compared to those of the three reference models, namely, STFT-CNN, CWT-CNN, and Prosvirin et al. [37], under different leak conditions, as shown in Figure 8, Figure 9 and Figure 10. The proposed method outperformed the reference models in all leak conditions, with an average ROC_AUC score of 0.4792 for leak conditions and a perfect score of 0.9998 for normal conditions. The reference (random) is shown with ROC_AUC 0.500.

**Figure 11:**
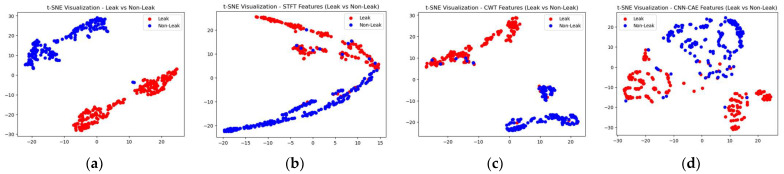
T-SNE visualization: comparative analysis of the (**a**) proposed model with (**b**) STFT-CNN, (**c**) CWT-CNN, and (**d**) Prosvirin et al. models (leak size = 1.0 mm).

**Figure 12:**
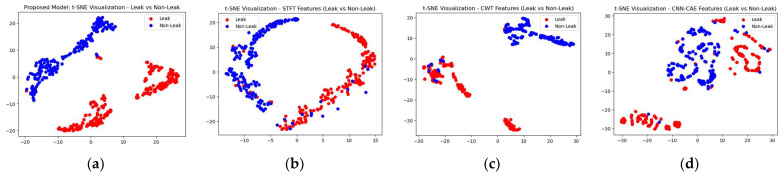
T-SNE visualization: comparative analysis of the (**a**) proposed model with (**b**) STFT-CNN, (**c**) CWT-CNN, and (**d**) Prosvirin et al. models (leak size = 0.5 mm).

**Figure 13:**
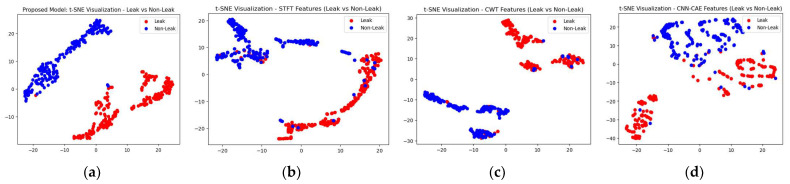
T-SNE visualization: comparative analysis of the (**a**) proposed model with (**b**) STFT-CNN, (**c**) CWT-CNN, and (**d**) Prosvirin et al. models (leak size = 0.3 mm).

**Figure 14:**
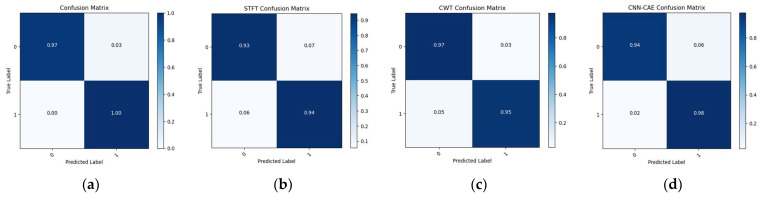
Confusion matrix comparison: (**a**) proposed model vs. (**b**) STFT-CNN, (**c**) CWT-CNN, and (**d**) Prosvirin et al. models (leak size = 1.0 mm).

**Figure 15:**
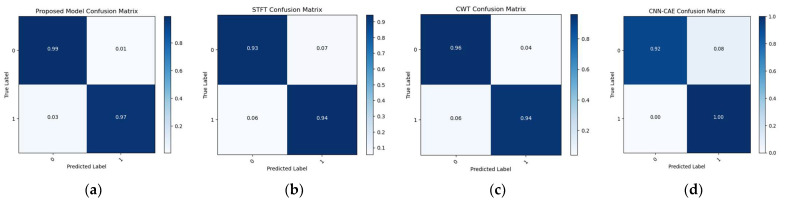
Confusion matrix comparison: (**a**) proposed model vs. (**b**) STFT-CNN, (**c**) CWT-CNN, and (**d**) Prosvirin et al. models (leak size = 0.5 mm).

**Figure 16:**
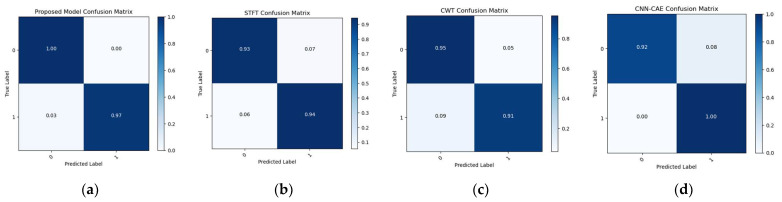
Confusion matrix comparison: (**a**) proposed model vs. (**b**) STFT-CNN, (**c**) CWT-CNN, and (**d**) Prosvirin et al. models (leak size = 0.3 mm).

To make the comparison more explicit, a comparison of t-SNE visualizations of the proposed and three reference models is shown in Figure 11, Figure 12 and Figure 13. These figures clearly illustrate that the proposed model outperformed the reference models in classification of the leak and normal conditions under different leak sizes (leak sizes of 1 mm, 0.5 mm, and 0.3 mm). Furthermore, Figure 14, Figure 15 and Figure 16 illustrate confusion matrix comparisons of the proposed model with all three reference models. The findings clearly indicate that the proposed model provides a comprehensive hybrid solution that is more effective and efficient for leak detection under different conditions.

## 6. Conclusions

In conclusion, pipeline leak detection is critical for maintaining the safety and integrity of fluid transportation systems. The timely and accurate identification of leaks is essential to prevent environmental harm, financial losses, and potential hazards to the public. However, relying solely on a single technique such as CWT for detecting pipeline leaks using AE signals has limitations. These include the trade-off between time and frequency resolution, the high dimensionality of CWT scalograms, and the need for careful selection of wavelet functions and parameters. It is crucial to adopt an integrated approach by combining complementary techniques such as CWT and STFT to address these challenges. By extracting features from CWT and STFT scalograms, a comprehensive representation of AE signals can be obtained, capturing a wide range of temporal and spectral details. The proposed hybrid model demonstrates superior performance, surpassing conventional models STFT-CNN and CWT-CNN, with an average accuracy of 99.22%. This approach provides a more accurate and reliable solution for real-time leak detection in various industrial applications.

In future research, an important focus should be placed on improving leak localization capabilities. While the proposed hybrid approach achieves high accuracy in leak detection, efforts should be directed toward developing methodologies that precisely identify the leak location along the pipeline. Integrating additional sensing technologies and advanced signal processing algorithms could enhance spatial resolution and enable precise localization. Future studies can advance pipeline monitoring and maintenance by further enhancing the hybrid model and exploring emerging technologies to ensure efficient leak detection and minimize risks associated with pipeline infrastructures.

## Figures and Tables

**Table 1:** Architecture overview of STFT-CNN Model and CWT-CNN Model.

Layer	Input Shape	No. of Filters	Kernel Size	Output Shape	Activation Function	Number of Parameters
Conv1	(3, 224, 224)	16	(3 × 3)	(16, 224, 224)	ReLU/-	448
Max Pooling	(16, 224, 224)	-	(2 × 2)	(16, 112, 112)	-	0
Conv2	(16, 112, 112)	32	(3 × 3)	(32, 112, 112)	ReLU/-	4640
Max Pooling	(32, 112, 112)	-	(2 × 2)	(32, 56, 56)	-	0
Conv3	(32, 56, 56)	64	(3 × 3)	(64, 56, 56)	ReLU/-	18,496
Max Pooling	(64, 56, 56)	-	(2 × 2)	(64, 28, 28)	-	0
Reshape	(64, 28, 28)	-	-	(50176)	-	0
FC1	(50176)	-	-	(128)	ReLU/-	6,407,040
FC2	(128)	-	-	(10)	-	1290

**Table 2:** Comprehensive details regarding the data-acquisition configuration and setup.

Datasets	Substance-Pressure (bar)	Leak Size (mm)	Duration (s)	Feature Vector Samples (Leak/Normal)
1	Water-13	1.0	360	240/120
2	Gas-13	0.5	360	240/120
3	Water-18	0.3	360	240/120
4	Gas-18	0.5	360	240/120

**Table 3:** Evaluation of model effectiveness: Proposed model versus STFT-CNN, CWT-CNN, and Prosvirin et al. models [37].

Models	Proposed			STFT-CNN			CWT-CNN			Prosvirin et al. [36]		
Leak Size	1 mm	0.5 mm	0.3 mm	1 mm	0.5 mm	0.3 mm	1 mm	0.5 mm	0.3 mm	1 mm	0.5 mm	0.3 mm
**Accuracy**	**99.74**	**98.70**	**99.22**	94.21	92.96	93.75	96.75	94.53	92.97	96.10	97.14	97.40
Avg. Accuracy	**99.22**			93.64			95.57			96.88		
**Precision**	**99.51**	**100**	**100**	94.22	93.04	93.76	97.12	95.05	94.44	97.14	96.10	97.22
Avg. Precision	**99.84**			93.67			96.50			96.82		
**F1 Score**	**100**	**98.75**	**99.26**	94.21	92.97	93.75	95.67	94.81	93.27	95.57	94.44	97.56
Avg. F1 Score	**99.34**			93.64			95.11			95.86		
**Recall**	**100**	**97.54**	**98.52**	94.21	92.97	93.76	95.93	94.58	92.12	94.44	95.57	97.56
Avg. Recall	**98.67**			93.65			95.78			95.86		

## Data Availability

The data were obtained from the industry. Owing to the privacy policy of the industry, the data are not publicly available.

## Nomenclature

ANN	artificial neural network
STFT	short-time Fourier transform
CWT	continuous wavelet transform
CNN	convolutional neural network
ReLU	rectified linear unit
DL	deep learning
AE	acoustic emission
CAE	convolutional autoencoder
EMD	empirical mode decomposition
VMD	variational mode decomposition
SVM	support vector machines
ROC	receiver operating characteristic
